# ^1^H-NMR analysis of feces: new possibilities in the helminthes infections research

**DOI:** 10.1186/s12879-017-2351-7

**Published:** 2017-04-17

**Authors:** Sarantos Kostidis, Daria Kokova, Natalia Dementeva, Irina V. Saltykova, Hye Kyong Kim, Young Hae Choi, Oleg A. Mayboroda

**Affiliations:** 1 Center for Proteomics and Metabolomics, Leiden University Medical Centre, Leiden, The Netherlands; 2 Department of Parasitology, Leiden University Medical Center, Leiden, The Netherlands; 3 Laboratory of clinical metabolomics, Tomsk State University, Tomsk, Russia; 4 Natural Products Laboratory, Institute of Biology, Leiden University, Leiden, The Netherlands; 5 Siberian State Medical University, Central Research Laboratory, Tomsk, Russia

## Abstract

**Background:**

Analysis of the stool samples is an essential part of routine diagnostics of the helminthes infections. However, the standard methods such Kato and Kato-Katz utilize only a fraction of the information available. Here we present a method based on the nuclear magnetic resonance spectroscopy (NMR) which could be auxiliary to the standard procedures by evaluating the complex metabolic profiles (or phenotypes) of the samples.

**Method:**

The samples were collected over the period of June-July 2015, frozen at −20 °C at the site of collection and transferred within four hours for the permanent storage at −80 °C. Fecal metabolites were extracted by mixing aliquots of about 100 mg thawed stool material with 0.5 mL phosphate buffer saline, followed by the homogenization and centrifugations steps. All NMR data were recorded using a Bruker 600 MHz AVANCE II spectrometer equipped with a 5 mm triple resonance inverse cryoprobe and a z-gradient system.

**Results:**

Here we report an optimized method for NMR based metabolic profiling/phenotyping of the stools samples. Overall, 62 metabolites were annotated in the pool sample using the 2D NMR spectra and the Bruker Biorefcode database. The compounds cover a wide range of the metabolome including amino acids and their derivatives, short chain fatty acids (SCFAs), carboxylic acids and their derivatives, amines, carbohydrates, purines, alcohols and others. An exploratory analysis of the metabolic profiles reveals no strong trends associated with the infection status of the patients. However, using the penalized regression as a variable selection method we succeeded in finding a subset of eleven variables which enables to discriminate the patients on basis of their infections status.

**Conclusions:**

A simple method for metabolic profiling/phenotyping of the stools samples is reported and tested on a pilot opisthorchiasis cohort. To our knowledge this is the first report of a NMR-based feces analysis in the context of the helminthic infections.

## Background

Analysis of stool samples is an essential part of routine diagnostics of the helminthes infections. For years, despite a consistent background of criticism and occasional new developments, the direct smear and Kato-Katz techniques remain the gold standard diagnostic tests for schistosomiasis, opisthorchiasis and the soil-transmitted helminthiasis [1]. However, here we introduce a method based on nuclear magnetic resonance spectroscopy (NMR), which could be auxiliary to the standard methodologies. In contrast to the Kato and Kato-Katz tests which use only the eggs count as a measure, we examine the complex metabolic profile of the sample. In other words we are applying the metabolomics approach. Metabolomics is a discipline studying the metabolome - a totality of the metabolites that can be measured in a biological sample. The metabolites are defined as the end products and the intermediates of the metabolism. In the clinical setting the metabolomics studies are commonly based on the analysis of the body fluids. Urine and blood (serum or plasma) are being the most common sample types due to the minimally invasive procedures of sample collection. Feces as a material for metabolomics studies has only recently started to gain the deserved attention [2, 3]. Over recent years few metabolomics studies in such areas as e.g. dietary interventions [4], inflammatory bowel disease [5, 6] and colorectal cancer [7] have been published.

Indeed, the fecal masses are the physiological product of the gastrointestinal tract, one of the key metabolic systems of the human body. Thus, it is logical to assume that their composition should reflect current metabolic status of the digestive tract or its metabolic phenotype [8]. The human gut represents a complex ecosystem and harbors gut bacteria outnumbering the cells in our organism [9] and the analysis of the fecal masses or/and their derivatives (e.g. extracts or fecal waters) offers the most direct access to the physiological processes controlling the gastrointestinal system homeostasis, gut bacteria-host interactions and interaction between the hosts and parasitic helminthes. For example, the helminth infections are often accompanied by such symptoms as diarrhea, abdominal pain and blood in the stool. The given examples represent the extreme cases, but they provide a clear illustration of the parasite’s ability changing the metabolic homeostasis of the host and the host’s digestive system in particular. This, in turn, makes metabolic analysis of the fecal masses an interesting, non-invasive way to monitor such changes.

Here we present a simple NMR based metabolomics workflow for the analysis of fecal samples. For this pilot study we used stool samples of patients diagnosed with opisthorchiasis and a group of matched controls. Opisthorchiasis is parasitic disease caused by trematodes belonging to the family Opisthorchiidae (*Opisthorchis felineus, Opisthorchis viverrini*) [10]. According to WHO there are about 17 million infected people and approximately 112 million people exposed or at risk of infection. The workflow presented here is only a proof of principle, but it can be easily scaled, tuned towards a quantitative analysis and implemented into other case studies or in future routine screening without fundamental modification of the sample collection or the exiting diagnostic routines.

## Methods

### Sample collection

The study was reviewed and approved by the local ethics committee of the Siberian State Medical University (Tomsk, Russia). The samples were collected over the period of June-July 2015. The samples were frozen at −20 °C at the site of collection and transferred within four hours for the permanent storage at −80 °C. The diagnosis of opisthorchiasis was confirmed by the Kato-Katz test [1]. Table 1 summarizes the demographic data of the patients. In total the samples of 30 patients (16 infected and 14 uninfected) were used.

**Table 1:** Characteristics of participants

Parameter	Summary	Opisthorchiasis (*n* = 16)	Control (*n* = 14)
Age (year)	range	(21, 64)	(24, 63)
	median	44	44
Gender	male	0	0
	female	16	14
BMI	range	(18.4, 43.6)	(21.7, 32.5)
	median	26.22	27.53

### Fecal metabolites extraction

Fecal metabolites were extracted as described elsewhere [11] with some minor modifications. Briefly, the aliquots of about 100 mg thawed stool material were mixed with 0.5 mL phosphate buffer saline (1.9 mM Na_2_HPO_4_, 8.1 mM NaH_2_PO_4_, 150 mM NaCl, pH 7.4; Sigma-Aldrich, Germany) containing 10% deuterated water (D_2_O 99.8%; Cortecnet, France) and 0.05 mM sodium 3-trimethylsilyl-propionate-*d*_4_ (TMSP-2,2,3,3-*d*_4_; Cambridge Isotope Laboratories Inc., UK) as chemical shift reference. The mixtures were homogenized by bead beating with zirconium oxide beads of 1 mm diameter for 30 s at 4 °C in a Bullet Blender 24 (Next Advance Inc., USA). The fecal slurry was then centrifuged at 16100×*g* for 15 min at 4 °C. Supernatants were collected and centrifugation was repeated. Finally, the resulting fecal extracts were transferred to a 96 well plate (Bruker, Germany) and 190 μL of each sample was transferred to a 3 mm NMR tube in SampleJet 96 tube rack (Bruker, Germany) using 215 Gilson liquid handler. The samples were then placed in a SampleJet system and kept cooled at 6 °C while queued for NMR measurements.

Alternative protocols for fecal extraction, as described elsewhere [5, 12, 13] were also applied using technical replicates and the same equipment and chemicals described above. For filtration we used the Whatman filters with 0.2 μm diameter pores (GE Healthcare, UK). An ultracentrifugation step with filtration was also tested using Amicon Ultra cellulose centrifugal filters with a cut-off MW of 3000 Da (Millipore Ireland, Ltd). The filters were washed with doubly distilled water before use and tested for impurities and presence of additives using a blank PBS buffer sample and acquisition of NMR spectra with the same parameters as those used for fecal extracts measurements (see below).

### NMR spectroscopy

All NMR data were recorded using a Bruker 600 MHz AVANCE II spectrometer equipped with a 5 mm triple resonance inverse cryoprobe and a z-gradient system. The temperature of the samples was controlled at 27 °C during measurement. Prior to data acquisition, tuning and matching of the probe head followed by shimming and proton pulse calibration were performed automatically for each sample. One-dimensional (1D) ^1^H NMR spectra were recorded using the first increment of a NOESY pulse sequence with presaturation (*γ*B_1_ = 50 Hz) for water suppression during a relaxation delay of 4 s and a mixing time of 10 ms [14, 15] 64 scans of 65,536 points covering 12,335 Hz were recorded and zero filled to 65,536 complex points prior to Fourier transformation, an exponential window function was applied with a line-broadening factor of 1.0 Hz. The spectra were automatically phase and baseline corrected and referenced to the internal standard (TMSP; *δ* 0.0 ppm).

After tube filling, 30 μL from the leftovers of each sample were combined to form a pool sample mix. The pool sample was aliquoted and used for acquisition of two-dimensional (2D) NMR spectra to aid the assignment of fecal metabolites. The set of 2D experiments included a *J*-resolve (*J*-res), ^1^H-^1^H correlation spectroscopy (COSY), ^1^H-^1^H total correlation spectroscopy (TOCSY), ^1^H-^13^C heteronuclear single quantum correlation (HSQC) and ^1^H-^13^C heteronuclear multiple bond correlation spectroscopy (HMBC) using the standard parameters implemented in Topspin 3.0 (Bruker Biospin, Germany).

### NMR data processing

NMR data were further processed using in house routines written in Matlab 2014a (The Mathworks, Inc., USA) and Python 2.7 (Python Software Foundation, www.python.org). Briefly, the obtained ^1^H spectra were re-evaluated for incorrect baselines and corrected using a polynomial fit of degree 5. The spectral region from 0.5 to 9.7 ppm was binned using an in-house algorithm for adaptive intelligent binning, which is based on the original paper of De Meyer et al. [16]. Initial bin width was set to 0.02 ppm and final variable bins sizes were calculated based on the peaks position and width in the spectra. The spectral region with the residual water peak (4.5 – 5.1 ppm) was excluded from the data. The final data consisted of 429 bins that were normalized by the Probabilistic Quotients Normalization method [17] to correct for dilution differences from sample to sample. Data were first normalized to unit total area and subsequently, the variables of each sample were divided by those of a reference sample, in this case the median spectrum. Each sample was subsequently scaled by its median quotient, which represents the most probable dilution factor. Finally, the normalized data was autoscaled prior to statistical analysis.

### Data analysis

All the analysis was performed in the R statistical software environment (http://www.r-project.org/, R version 3.2.3.). Exploratory data analysis was performed using the package “pcaMethods” [18]. Variable selection was performed with the “glmnet” package [19]. For data visualization the “ggplot2”, “GGally” and “gridExtra” packages were used.

## Results

### Optimization of the sample preparation

**Fig. 1:**
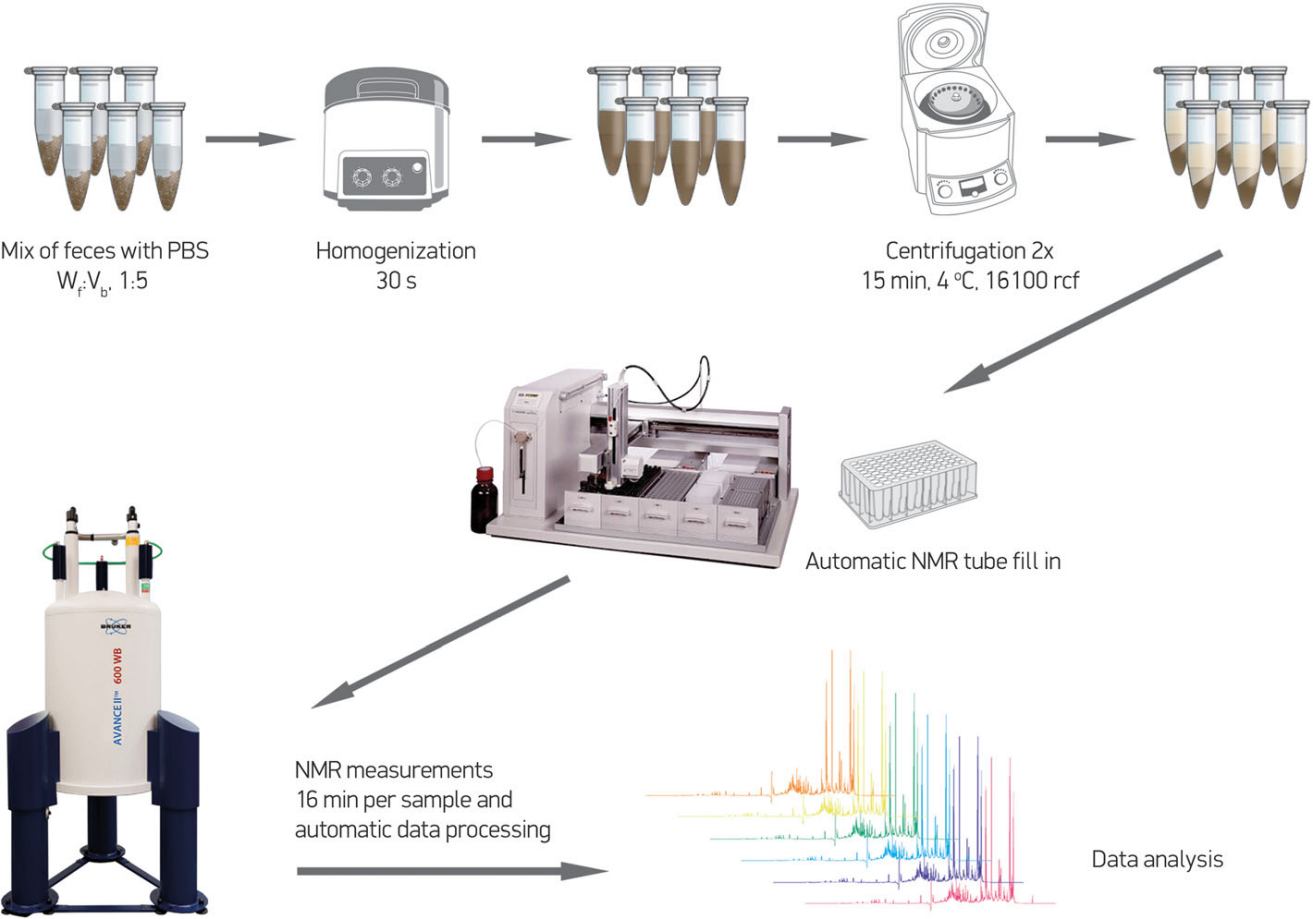
A schematic outline of the sample preparation workflow

In contrast to other body fluids like urine and blood for which the well-established standard operating procedures (SOP) exist, no consensus for feces handling has been reached yet. Thus, to get an optimal extraction of the feces samples several protocols described in the literature [11–13] were tested. A detailed overview of the available methods can be found in recent review by Deda et al. [2]. In our case, a minimally modified protocol of the one recently suggested by Lamichhane et al. [11] provided an optimal outcome in terms of spectra quality and the number of the metabolites detected. In the original manuscript, the authors suggested mixing the fecal material with 2 volumes of PBS (W_f_:V_b_; mg of feces x μL^−1^ of PBS buffer), which according to them provides better signal to noise ratios and minimal compromise for peak shifting due to small inter-sample pH differences. They also used a freeze-thaw cycle with centrifugation of the fresh fecal slurry, storage at −80 °C and thawing at the day of analysis followed by a second centrifugation. Since, the samples used in our study were already frozen and stored at −80 ^°^C at the site of collection we opted to avoid the extra freeze-thaw step. Therefore, after thawing the frozen fecal aliquots and the homogenization of the fecal slurry, we performed two consecutive centrifugation steps at 4 ^°^C. The suggested 1:2 W_f_:V_b_ ratio did not work well in our case as the supernatants could not be easily separated from the precipitated material even after extending the centrifugation time. An obvious solution would be to include a filtration step but this would require an extra step to wash the filters, which increases the time and costs of the protocol. On the other hand, we found that by using the 1:5 W_f_:V_b_ and 7 min 1D ^1^H-NMR acquisition method (64 scans per sample) the losses in the signal to noise ration were minimal even for the weak signal of formic acid (SNR 36.3 and 29.8 for 1:2 and 1:5 W_f_:V_b_, respectively) while the peak shifting of pH sensitive protons was reduced comparing to 1:2 W_f_:V_b_ as an effect of better pH control. We therefore decided to follow the 1:5 W_f_:V_b_ mixing with PBS for all the samples analyzed in this study. Figure 1 shows a schematic representation of the entire workflow.

**Fig. 2:**
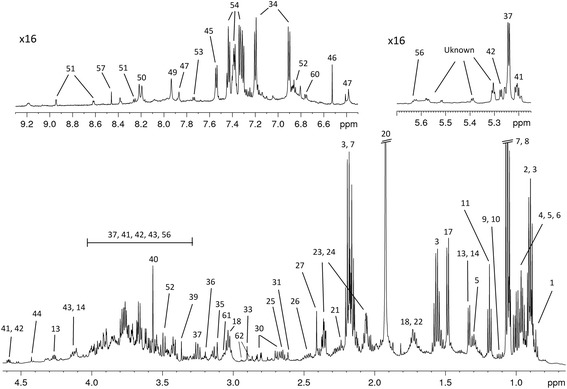
Regions of the 600 MHz 1D ^1^H NMR spectrum of the pool sample mix of all fecal extracts used in this study. The regions on top are multiplied 16 times for better visualization. 60 fecal metabolites were identified with most of them annotated on the spectrum. Metabolites and their numbering as displayed in figure: 1: 2-methylbutyrate; 2: Valerate; 3: n-butyrate; 4:Leucine; 5:Isoleucine; 6: Valine; 7:Propionate; 8: Isobutyrate; 9: 3-methyl-2-oxoisovalerate; 10: 2-oxoisovalerate; 11: Ethanol; 12: 3-hydroxybutyrate; 13: Threonine; 14: Lactate; 15: 2-hydroxyisobutyrate; 16: 3-hydroxy-2-butanone; 17: Alanine; 18: Lysine; 19: Thymine; 20: Acetate; 21: 5-aminopentanoate; 22: Ornithine; 23: Proline; 24: Glutamate; 25: Methionine; 26: Glutamine; 27: Succinate; 28: 2-oxoglutarate; 29: 3-phenylpropionate; 30: Aspartate; 31: Methylamine; 32: Malate; 33: Trimethylamine; 34: Tyrosine; 35: Malonate; 36: Choline; 37: D-glucose; 38: Taurine; 39: Methanol; 40: Glycine; 41: D-xylose; 42: D-galactose; 43: Fructose; 44: Dihydroxyacetone; 45: Uracil; 46: Fumarate; 47: Urocanate; 48: Ethanolamine; 49: Xanthine; 50: Hypoxanthine; 51: Nicotinate; 52: 3-hydroxyphenylacetate; 53: Tryptophan; 54: Phenylalanine; 55: Orotate; 56; UDP-glucuronate; 57: Formate; 58: Benzoate; 59: 4-aminohippurate; 60: Homovanillate; 61: Putrescine; 62: Asparagine

Figure 2 shows the ^1^H spectrum of a pooled sample with annotations of the identified metabolites. Overall, 62 metabolites were annotated in the pool sample using the 2D NMR spectra and the Bruker Biorefcode database (Bruker Biospin, Germany). The detected compounds cover a wide range of the metabolites including amino acids and their derivatives, short chain fatty acids (SCFAs), carboxylic acids and their derivatives, amines, carbohydrates, purines, alcohols and others. The complete list of metabolites is enumerated in the legend of Fig. 2.

### Exploratory analysis of the data

**Fig. 3:**
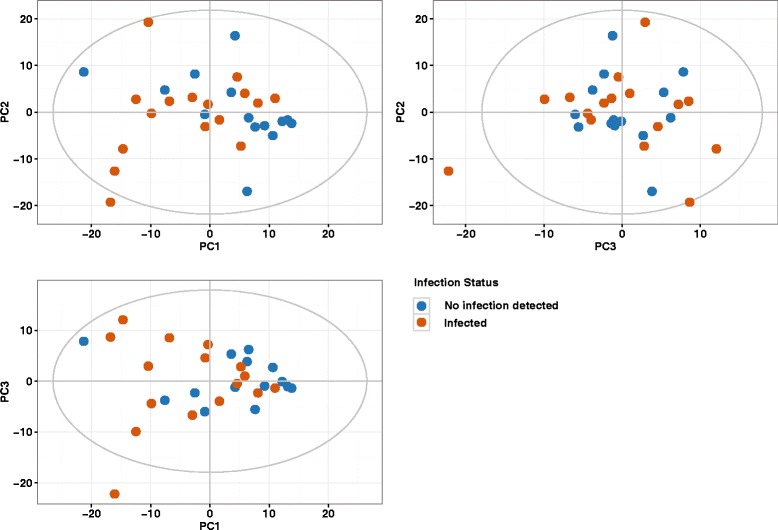
PCA score plots for the first three components

The main purpose of an exploratory data analysis is to reveal the major trends in the data as well as the possible analytical and/or biological confounders if any. Principal Component Analysis (PCA) is commonly used method for such analysis. Figure 3 shows a combined score plot of the first three principal components of the PCA model. The first three components cover almost 50% (~ 49) of the total variance in the data but apparently the infection status does not represent a visible trend in the data. Since initial PCA model failed to describe any tendencies in the data associated with the study design we built a two-class Partial Least Squares Discriminant Analysis (PLS-DA) model with infections status as a class ID. The model proved to be a statistically poor and described the data narrowly better than a random one (data not shown). One could interpret the results as a lack of association between infection status and metabolic composition of the feces. The performance of the PLS-DA model clearly supports such interpretation. However, the structure of our data set (30 observations and 429 variables) is such that the number of predictive variables (p) is much larger than the number of samples (n). The PLS-DA method, despite being one of the most popular classification methods in metabolomics analysis, is a suboptimal choice for the p> > n data sets [20]. Thus, we decided to employ an alternative data analysis strategy including a variable selection step which could identify a subset of predictors relevant to the study design.

### Variable selection and validation of the selected subset

**Fig. 4:**
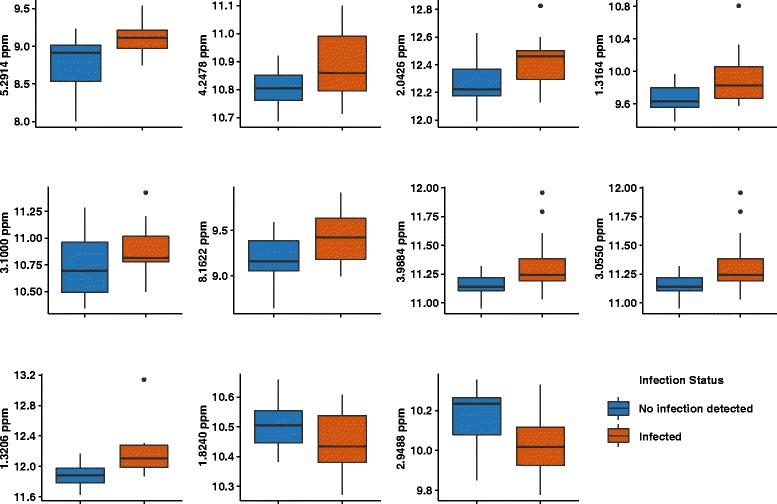
Box-plots for the variables selected with the lasso regression. The variable assignments and corresponding *p*-values are shown in the Table 2

The analysis of high dimensional datasets has progressed enormously since the beginning of “omics” era. Several methods specifically addressing p> > n problem are described and tested in practice, but the area of application for the methods is mainly restricted to the genomics data [21, 22]. We deiced to use the penalized regression approach based on its “track record” in solving comparable problems, namely a high number of the variables and the limited number of the observations [22, 23]. A penalized variable section belongs to the class of the regulations methods: the methods which improve the estimates “for over-parameterized problems through the use of additional assumptions, prior information or penalties” [24]. A subset of eleven variables was selected using a lasso type of penalty. Before subjecting the set of selected variables to the next statistical test we have also made an inventory of the selection trying to estimate whether the feature selecting routine has picked the NMR spectral areas influenced by the noise and/or any baseline effect. Figure 4 shows the box plots for all eleven predictors selected by this method. Table 2 summarizes all the selected bins showing their corresponding spectral regions, identity and Benjamini-Hochberg corrected *p*-values. Finally, we have included the selected variables into a logistic regression model. The resulting model is characterized by the chi square 27.74 and chi square probability of 1.05E-4.

**Table 2:** The selected variable assignments and corresponding p-values

Spectral region (ppm)	*p*-value	ID
5.2914	0.0378	Unknown triplet/4.12/1.99/1.80
4.2478	0.0942	Threonine
2.0426	0.0601	Glutamate/Proline/Isovalerate
1.3164	0.0378	Threonine and Lactate
3.1000	0.1661	Malonate
8.1622	0.0378	Hypoxanthine
3.9884	0.0601	Phenylalanine / Fructose
3.0550	0.0941	Tyrosine / Ornithine / Putrescine
1.3206	0.0031	Threonine and Lactate
1.8240	0.1322	Ornithine / Unknown
2.9488	0.0378	Asparagine

Benjamini/Hochberg test for multiple testing correction was used to adjust *p*-values

## Discussion

Here we present an analytical workflow for ^1^H-NMR analysis of feces with special emphasis on application in the field of the helminthes infections. The described procedure resulted in rich spectra where 62 metabolites are annotated (Fig. 2). Using our set of the samples selection we were able to dissect a subset of the metabolites (Fig. 4) which may be discriminative for the infections status. This subset includes such common constituents of human biofluids as threonine, asparagine, lactate and hypoxanthine. Asparagine is higher in the samples of the control patients while the other selected compounds have higher levels in the infected samples. The limited number of samples is a clear limitation of this study and therefore we restrain ourselves from the discussion of the possible physiological models based on the selected markers or the attempts to deconvolute the metabolic profiles into the infection predictive patterns. On the other hand the proposed method clearly stresses out the potential for a new window of information that can be used in such case studies. In principle, the fact that a subset of the discriminative metabolites can be dissected gives a clear illustration of the method’s potential. A combination of a simple, commonly accepted diagnostic method and such advanced analytical method as NMR provides a powerful research tool which enables the collection of a wealth of information without interference or in parallel with the routine diagnostics or epidemiological studies. Taking advantage of the robustness and quantitative nature of this technology, obtaining the metabolic profiles of fecal material is rather straightforward and provides both an insight into biochemistry/physiology of the host-pathogen interaction and the possibility of accessing the morbidity and eventually play an auxiliary role in the diagnostics. The main limitations of this approach arise mainly from the absence of standard procedures in stool collection rather than the technology itself. However, taking into account the increasing interest in using the NMR (as well as mass spectrometry) based metabolomics approaches in fecal samples, we envisage that more established routines and practices in sample collection will be developed in the near future which will reveal the underlying potential of this type of analysis.

## Conclusions

In summary, a simple method for metabolic profiling/phenotyping of the stools samples is reported and tested on a pilot opisthorchiasis cohort. To our knowledge this is the first report of a NMR-based feces analysis in the context of the helminthic infections. With this study, an attempt was made to extend a conventional way of the stool analysis adding an extra dimension which can be used for metabolic phenotyping of the patients, in depth exploration of the host-parasite interaction and search for metabolic morbidity or/and infection markers. To extend and take full advantage of the possibilities offered by NMR based metabolic profiling much larger cohorts than the one used in this study are needed, preferably, even collected in the different endemic areas. With this report however, we provide a simple proof of concept aiming to introduce a well-established technology in the field of infectious diseases and fecal material analysis and with this, trigger future studies in this direction.

## Abbreviations

COSY: ^1^H-^1^H correlation spectroscopy
HMBC: Heteronuclear multiple bond correlation spectroscopy
NMR: Nuclear magnetic resonance
PCA: Principal Component Analysis
PLS-DA: Partial Least Squares Discriminant Analysis
SOP: standard operating procedures
TOCSY: ^1^H-^1^H total correlation spectroscopy
